# The Impact of Short, Structured ENT Teaching Interventions on Junior Doctors’ Confidence and On-Call Preparedness: A Systematic Review

**DOI:** 10.3390/healthcare13222886

**Published:** 2025-11-13

**Authors:** Mohammed Hasan Al-Khafaji, Ali Alabdalhussein, Shahad Al-Dabbagh, Abdulmohaimen Altalaa, Ghaith Alhumairi, Zeinab Abdulwahid, Anwer Al-Hasani, Juman Baban, Mohammed Al-Ogaidi, Eshtar Hamid, Manish Mair

**Affiliations:** 1 Department of Otolaryngology, Sherwood Forest Hospitals NHS Foundation Trust, Nottingham NG17 4JL, UK; mhkhachi@doctors.org.uk; 2 Department of Otorhinolaryngology, University Hospitals of Leicester NHS Foundation Trust, Leicester LE1 5WW, UK; ali.alabdalhussein@nhs.net; 3 Department of Psychiatry, Leicestershire Partnership NHS Trust, Leicester LE3 9EJ, UK; shahad.aldabbagh@doctors.org.uk; 4 Department of Acute Medical Care, East Lancashire Hospitals NHS Trust, Blackburn BB2 3HH, UK; abdulmohaimen.altala@elht.nhs.uk (A.A.); zeinab.abdulwahid@elht.nhs.uk (Z.A.); 5 Department of Medicine, Royal Stoke University Hospital, Stoke-on-Trent ST4 6QG, UK; ghaith.alhumairi@nhs.net; 6 Park Lane Surgery General Practice, Porth CF39 8AG, UK; anwer.al-hasani@wales.nhs.uk; 7 Department of Medicine, West Middlesex University Hospital, London TW7 6AF, UK; juman.baban@nhs.net; 8 Department of Haematology, University Hospital Southampton NHS Foundation Trust, Southampton SO16 6YD, UK; mohammed.al-ogaidi@uhs.nhs.uk; 9 School of Medicine, Faculty of Health, Medicine and Social Care, Anglia Ruskin University, Chelmsford CM1 1SQ, UK; eshtar.hamid@aru.ac.uk; 10 Department of Maxillofacial Surgery, University Hospitals of Leicester NHS Foundation Trust, Leicester LE1 5WW, UK

**Keywords:** otolaryngology, simulation-based education, bootcamps, junior doctors, on-call preparedness, e-learning

## Abstract

**Highlights:**

**What are the main findings?**

- Short, structured ENT teaching (boot camps/simulation/workshops) consistently improved junior doctors’ immediate confidence and knowledge; two studies also showed gains on blinded objective performance.
- The evidence is moderate quality (mean MERSQI 10.0) and largely single-group pre–post with limited follow-up, constraining causal inference and retention claims.

**What are the implications of the main findings?**

- Services can implement a three-arm programme—e-learning (core knowledge), case-based discussions (decision-making), and simulation (hands-on skills)—incorporating observed assessment and a defined core outcome set.
- Future studies should use comparative designs with blinded scoring and 2–3-month follow-up to evaluate behaviour/clinical impact (Kirkpatrick 3–4) and report instrument validity.

**Abstract:**

Background/Objectives: Ear, nose, and throat (ENT) presentations are common across the UK healthcare system and are often managed initially by junior doctors on call. Short, structured teaching interventions (e.g., boot camps and simulation workshops) have been introduced to improve confidence and preparedness. This review evaluated evidence published since 2015 on such ENT teaching interventions for junior doctors, examining effectiveness, study design, and outcome measures. Methods: Five databases were searched (January 2015–July 2025). Eligible studies assessed ENT-specific courses for junior doctors and reported outcomes on confidence, preparedness, knowledge, or performance. Study quality was appraised using the Medical Education Research Study Instrument (MERSQI). Owing to heterogeneity, findings were narratively synthesised in line with Synthesis Without Meta-analysis (SWiM) guidance. Results: Eleven studies (*n* = 591) met inclusion criteria: nine single-group pre–post studies, one two-group comparative study, and one randomised controlled trial (RCT). Most studies reported increased confidence after the interventions, while three also showed gains in knowledge. A minority reported improvement using blinded performance assessments. Overall methodological quality assessed using MERSQI scores was moderate (mean 10.0/18). Limitations included reliance on self-reported outcomes, limited use of control groups, and generally short follow-up periods. Conclusions: Short, structured ENT courses for junior doctors are associated with immediate improvements in confidence and knowledge, with some evidence of objective performance gains. However, the predominance of single-arm designs and brief follow-up limits causal inference and conclusions regarding retention, workplace behaviour, and patient outcomes. More robust comparative studies with blinded assessment and longitudinal follow-up are needed to determine sustained impact.

## 1. Introduction

Ear, nose and throat (ENT) emergencies are a recurring challenge across the UK healthcare system [1]. They present not only to ENT teams but also to Accident and Emergency departments, general practice, and other inpatient specialties, with ENT presentations accounting for around ten per cent of GP consultations [2]. Therefore, initial management of these presentations falls to junior doctors on call who face ENT-specific problems such as severe epistaxis, peritonsillar abscess, and airway compromise. This early decision-making carries risk if mismanaged, as surveys report that many feel anxious and underprepared to deal with these cases, particularly during unsupervised, out-of-hours work [1,3,4].

Many junior doctors feel underprepared for ENT on-call duties, as shown in UK surveys [3] and qualitative work [5]. This is linked to limited undergraduate exposure to ENT. Despite frequent ENT presentations across the specialties that junior doctors rotate through, the undergraduate exposure is limited to 1–2 weeks and, in some schools, none at all [6,7]. This mismatch contributes to a transition gap at the start of postgraduate training.

In response, a variety of short, structured teaching interventions have emerged to bridge the training gap. These include bootcamps, simulation workshops, and blended e-learning courses, all aimed at enhancing junior doctors’ confidence, technical skills, and clinical decision-making [1,4]. Across studies, short-term benefits are reported consistently, and in stronger designs, there are objective gains, for example, higher blinded viva scores [8] and improved performance on video-rated scenarios [9]. Nevertheless, content and evaluation vary widely, ranging from e-learning modules [10] and blended teaching formats [11] to high-fidelity simulations [12,13], so the landscape remains fragmented, with limited standardisation [1] and variable methodological quality [14,15], making it difficult to identify best practice or assess overall effectiveness or judge generalisability.

A critical limitation of the current evidence base is its heavy reliance on self-reported confidence as a primary outcome, rather than behaviour or patient outcomes [16]. Many studies lack control groups, which in turn limits causal inference in pre–post designs [17]. Most included studies in this review used single-group pre–post designs, with no or limited follow-up, so retention and behaviour change are unclear. Because confidence is not competence, and may be overestimated immediately after a course [18], validated knowledge tests and observed performance measures, ideally at the “shows how/does” levels, are recommended [19].

To address this evidence gap, this review seeks to answer the following key questions:

1. What types of short, structured ENT teaching interventions have been implemented for junior doctors since 2015?
2. How effective are these interventions in improving confidence, knowledge, and preparedness for ENT on-call responsibilities?
3. What insights from current interventions can inform the development of a scalable, standardised teaching framework for postgraduate ENT training?

This systematic review therefore addresses a clear gap by synthesising the effectiveness and design features of the available evidence on short, structured ENT educational interventions post-2015. It goes beyond asking whether they improve confidence by examining design and outcome measures. The ultimate goal is to inform a more standardised and evidence-based approach to ENT training, rather than one based on confidence alone.

### 1.1. Aim

To synthesise the available evidence of the effectiveness of short, structured ENT teaching interventions aimed at improving junior doctors’ confidence and preparedness for on-call ENT responsibilities.

### 1.2. Objectives

1. To systematically identify and describe short, structured ENT induction/teaching interventions for junior doctors implemented since 2015.
2. To synthesise evidence on confidence, knowledge, and observed performance (‘shows how’/‘does’), including follow-up and instrument validity reporting.
3. To propose a scalable three-arm framework (e-learning, interactive cases, simulation) and priority evaluation methods (comparative designs, blinded assessment, 2–3-month follow-up, core outcomes).

## 2. Materials and Methods

### 2.1. Study Design

A systematic review was conducted to evaluate the effectiveness of short, structured ENT teaching interventions in improving junior doctors’ confidence and on-call preparedness. This design was chosen to ensure a rigorous, transparent, and reproducible synthesis of existing evidence, in line with narrative synthesis guidance outlined by Popay et al. [20]. This approach is crucial for identifying effective educational components and informing future curriculum development.

### 2.2. Research Question and Framework

This systematic review addressed the following research question: “How do short, structured ENT teaching interventions affect junior doctors’ confidence and preparedness for ENT on-call responsibilities?”

To guide the review protocol and ensure a structured search and selection process, the question was formulated using the PICOS framework (Population, Intervention, Comparison, Outcomes, Study design), originally described by Richardson et al. [21]. The review followed the reporting principles outlined in the PRISMA 2020 statement [22], which provides updated guidance for the transparent reporting of systematic reviews.

### 2.3. Search Strategy

A comprehensive literature search was performed across five electronic databases: PubMed, MEDLINE (Ovid), EMBASE, CINAHL Plus, and the Cochrane Library, covering publications from January 2015 to July 2025. The search strategy employed the following Boolean search string across all databases:

(“bootcamp” OR “simulation” OR “teaching intervention” OR “structured course” OR “workshop”) AND (“ENT” OR “ear nose throat” OR “otolaryngology”) AND (“junior doctor” OR “foundation doctor” OR “SHO” OR “trainee doctor*”) AND (“confidence” OR “preparedness” OR “competence”).

Searches were restricted to peer-reviewed articles published in English. Screening, including duplicate removal, title, and abstract assessment, was facilitated using Rayyan software (2025) [23].

### 2.4. Study Selection

Study selection was conducted in accordance with PRISMA 2020 guidelines (Supplementary File: PRISMA 2020 checklist) [22]. All identified records were uploaded to Rayyan, where duplicate records were identified and removed [23]. Titles and abstracts were screened independently by two reviewers, with consensus reached through discussion. Full-text screening of eligible studies was then conducted to assess alignment with the predefined inclusion and exclusion criteria.

- Inclusion criteria: ENT-specific short courses for junior doctors, reporting outcomes on confidence, preparedness, knowledge, or competence, published between 2015–2025.
- Exclusion criteria: Studies focused solely on undergraduate medical students, interventions where ENT was a minor or embedded component of a broader programme, editorials, opinion pieces, and studies lacking defined methodologies or measurable outcome data.

### 2.5. Critical and Ethical Appraisal

The methodological quality of the included studies was assessed using the Medical Education Research Study Quality Instrument (MERSQI), a validated tool designed to evaluate the rigour of quantitative studies in medical education across six domains: study design, sampling, type of data, validity, data analysis, and outcomes [24,25]. To enhance reliability, MERSQI scoring was independently performed by two raters, followed by consensus discussion and justification for each score. Agreement was reached on all final scores through evidence-based deliberation. Inter-rater agreement across the 66 domain-level MERSQI ratings was 93.9%, with discrepancies discussed and resolved through consensus. In addition, the ethical conduct of each study was appraised by checking whether ethical approval and participant consent were reported.

### 2.6. Risk of Bias Appraisal

In addition to MERSQI scoring, a proportionate risk of bias (RoB) appraisal was conducted to complement the methodological quality assessment and enhance transparency regarding study-level internal validity. Non-randomised studies were assessed using the ROBINS-I tool [26], while the single randomised controlled trial was evaluated using the RoB 2.0 tool [27]. Given that MERSQI already captures core aspects of study design, sampling, data validity, and analytical rigour in medical education research, the RoB assessment was intentionally applied in a light-touch, context-sensitive manner.

The resulting judgements are summarised in Supplementary Table S1 and are not discussed in detail within the main text, to maintain focus on methodological quality appraised via MERSQI.

### 2.7. Data Extraction and Synthesis

A standardised data extraction form was used to record key variables from each study, including author, year, study design, intervention details, participant characteristics, and reported outcomes. Due to the significant heterogeneity in intervention formats, outcome measures, and study designs, a meta-analysis was not feasible. Therefore, a narrative synthesis approach was employed to thematically analyse and summarise the findings, in accordance with established guidance [20] and reported in line with SWiM guidance [28].

### 2.8. Ethical Approval and Registration

This review used only published, anonymised data and involved no human participants; therefore, ethical approval and consent were not required. The review was retrospectively registered on the Open Science Framework (OSF) (Registry ID: x2cvm; Registered 29 October 2025). No amendments to the registered methodology were made after registration.

## 3. Results

The results are presented in four parts: study selection, study characteristics, methodological quality, and outcome synthesis across confidence, knowledge, observed performance, and learner satisfaction.

### 3.1. Study Selection

**Figure 1:**
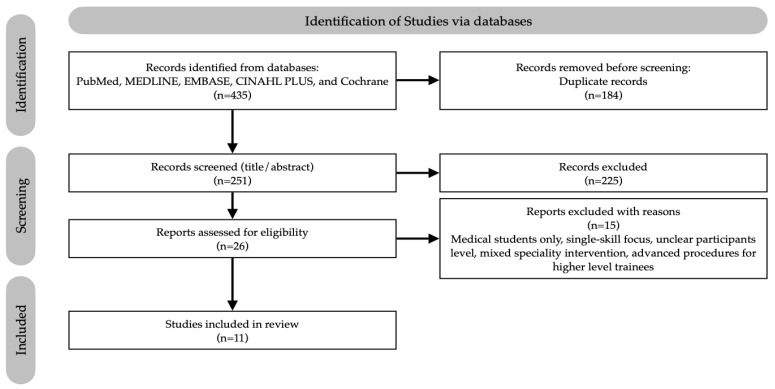
PRISMA 2020 flow diagram of study selection.

The PRISMA flow diagram (Figure 1) summarises the search and selection process [22]. In total, 435 records were identified. After removing 184 duplicates, 251 titles/abstracts were screened. Twenty-six full-text articles were assessed for eligibility; fifteen were excluded for different reasons (e.g., undergraduate-only populations, single-skill focus, unclear participant level, or targeting higher-level trainees). Eleven studies met all criteria and were included in the review. All included studies were screened for potential overlapping samples based on author, institution, cohort characteristics, and intervention date; no overlap was identified.

### 3.2. Study Characteristics

The 11 included studies (published 2015–2025) enrolled 591 participants, comprising foundation doctors, core surgical trainees, and ENT residents; five were UK-based (plus one from Ireland). Most interventions were short, single- or two-day intensive bootcamps or workshops centred on otolaryngology emergencies, using simulation from low-fidelity task trainers to high-fidelity team scenarios. Nine studies employed a single-group pre–post design, one used a two-group comparative design, and one was a randomised controlled trial. The most common outcomes were self-assessed confidence/competence (10/11 studies); three studies reported knowledge gains on MCQ tests, and one RCT used a blinded viva as the primary outcome. Key characteristics are summarised in Table 1.

### 3.3. Methodological Quality and Key Findings

The methodological quality and key findings are summarised in Table 2. Using the MERSQI tool [25], total scores ranged from 7.5 to 13.5 (mean 10.0/18), indicating moderate overall quality. The randomised controlled trial by Smith et al. (2015) [8] achieved the highest score (13.5) owing to its rigorous design, control arm, and blinded, objective performance outcome. Full item-level MERSQI scoring with detailed justification for each included study is provided in Table S2 (Supplementary Materials).

Common strengths included high response rates and use of inferential statistics. Although some studies recruited trainees from multiple programmes, MERSQI’s sampling domain counts implementation site(s) rather than the diversity of participating institutions; thus, courses delivered at a single host site (e.g., Cervenka et al., 2020 [13] at UC Davis) are scored as single-institution delivery. Recurrent limitations were heavy reliance on self-reported outcomes (10/11 studies; only Giri et al., 2024 [12] used objective MCQs alone); absence of control groups in most studies (9/11), restricting causal inference [17]; and inconsistent reporting of instrument validity and reliability, limiting interpretability and generalisability. A summary of risk-of-bias judgements across included studies is provided in Supplementary Table S1.

### 3.4. Synthesis of Results

The synthesis of results is structured around the primary outcome types identified across the studies.

#### 3.4.1. Self-Reported Confidence and Competence

Ten of eleven studies that assessed self-reported confidence or competence reported statistically significant post-intervention improvements. For example, Jegatheeswaran et al. (2023) [29] found significant gains across all seven core ENT skills (*p* < 0.001). Bhalla et al. (2020) [10] provided comparative evidence: confidence increased significantly in the simulation group (*p* < 0.001), whereas the lecture-only group showed no significant change. The exception was Giri et al. (2024) [12], which focused solely on objective knowledge through an MCQ assessment and did not measure self-reported confidence.

#### 3.4.2. Knowledge Acquisition

Three studies by Morris et al. (2025) [30], Bhalla et al. (2020) [10], and Giri et al. (2024) [12] used multiple-choice questions (MCQs) to assess knowledge acquisition, all reporting statistically significant post-intervention improvements. Morris et al. (2025) [30] reported a substantial improvement in knowledge scores, with participants’ mean scores rising from 68.5% to 96.5% (*p* < 0.01) following the bootcamp. Bhalla et al. (2020) [10] found that at one-month follow-up, the simulation group retained significantly more knowledge (mean score: 17/20) than the lecture-only group (12.3/20). Giri et al. (2024) [12] also reported a significant gain in MCQ scores after a didactic ENT workshop (*p* < 0.0001) that did not incorporate simulation, highlighting the potential value of structured didactic training.

#### 3.4.3. Clinical Performance

Two studies used objective, blinded performance assessments. Swords et al. (2017) [9] reported significant improvement on blinded video-rated scenarios (mean 9.75→18.75/30; *p* = 0.0093). In the only RCT, Smith et al. (2015) [8] found higher blinded viva scores in the simulation group compared with the lecture-only group (*p* < 0.05).

#### 3.4.4. Learner Satisfaction and Perception

All studies that evaluated satisfaction reported positive feedback. Examples include Dell’Era et al. (2020) [32] (median SSES 4.5/5), Jegatheeswaran et al. (2023) [29] (100% recommendation), and Morris et al. (2025) [30] (high satisfaction); similar patterns were noted in Chin (2016) [11], Cervenka (2020) [13], La Monte (2023) [31], and Alabi (2022) [33].

Collectively, the studies show consistent gains in confidence, knowledge, and learner satisfaction, alongside limited but encouraging evidence of objective performance improvement.

## 4. Discussion

### 4.1. Summary of Major Findings

This systematic review examined short, structured teaching interventions designed to prepare junior doctors and early trainees for ENT placements and on-call emergencies. Across eleven studies, simulation bootcamps and practical workshops were consistently associated with short-term gains in self-reported confidence and, in several studies, knowledge. In stronger designs, objective performance gains were demonstrated—for instance, Smith et al. (2015) [8] reported higher scores on a blinded viva in the simulation arm, while Swords et al. (2017) [9] observed improved performance in blinded assessments of video-recorded scenarios. Additionally, three studies—by Bhalla et al. (2020) [10], Giri et al. (2024) [12], and Morris et al. (2025) [30]—reported significant improvements in knowledge using multiple-choice tests. Notably, Bhalla et al. (2020) [10] also demonstrated a short-term retention advantage for simulation over lecture-only teaching.

These findings are promising but should be interpreted with caution. Most studies relied on self-reported confidence, used single-group pre–post designs, and seldom included longer-term follow-up or knowledge refreshers. Validity evidence for outcome instruments was inconsistently reported across studies, and intervention formats varied widely. Altogether, these issues limit causal inference [17] and make it difficult to determine whether short-term learning lasts, translates into behaviour change in practice, and eventually improves patient outcomes. Accordingly, more high-quality comparative studies with blinded, objective assessment and longitudinal follow-up are needed.

### 4.2. Interpretation in the Context of Existing Literature

These findings are consistent with wider evidence from health professions education. Simulation offers safe, supported practice with structured feedback and clear pass standards [34], leading to significant gains in knowledge and skills [35]. A multimodal design—e-learning for foundational knowledge followed by simulation and case discussion—is therefore well-supported. This structure not only aligns with the cognitive theory of multimedia learning [36] but is also supported by evidence showing that e-learning and blended formats perform at least as well as traditional teaching for knowledge acquisition [37,38].

**Figure 2:**
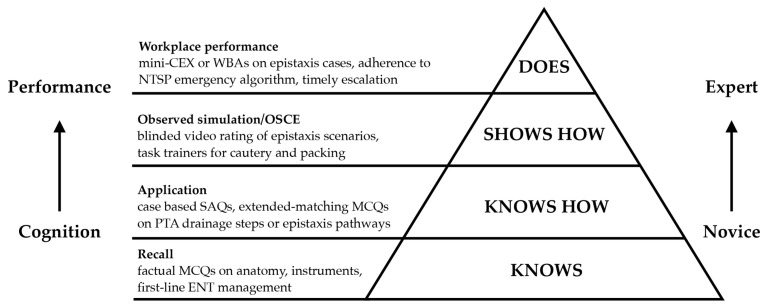
Miller’s pyramid of clinical competence with ENT-specific assessment examples. Redrawn and adapted by the authors from Miller (1990) [19]. mini-CEX: Mini Clinical Evaluation Exercise, WBAs: Workplace-Based Assessments, NTSP: National Tracheostomy Safety Project, SAQs: Short-Answer Questions, MCQs: Multiple-Choice Question(s), PTA: Peritonsillar abscess.

These patterns are also credible on learning theory grounds. Deliberate practice with feedback-rich tasks and clear pass standards helps explain the gains seen at simulation stations for tasks like epistaxis control or tracheostomy emergencies—a finding supported by evidence from the simulation literature [34] and the foundational theory of deliberate practice [39]. Cognitive load theory supports chunked steps, close guidance, and graded complexity, which is why focused skills stations tend to outperform passive lectures for procedures such as nasal cautery or peritonsillar abscess drainage [40,41]. Experiential learning fits the bootcamp flow of do, debrief, consolidate, and retry [42]. Framed by Miller’s pyramid, most outcomes here sit at ‘knows’ or ‘shows how’ [19], so assessment should push toward observed performance that approximates ‘does’ (see Figure 2). Set against this, confidence is not competence. Many outcomes sit at Kirkpatrick Levels 1–2 and rely on self-report [16]. Moreover, novices can overestimate after a course, so programmes should pair confidence checks with validated knowledge tests and observed, ideally blinded, performance measures where feasible [18]. Frequent low-stakes testing supports retention and transfer, so routine retrieval practice should sit alongside simulation [43].

Most UK medical schools offer only brief ENT placements, sometimes none at all, so the baseline is uneven before postgraduate training even begins [6,7]. As a result, induction and preparedness vary significantly across trusts [3]. The strongest evidence supports structured practice with feedback—therefore, we propose a three-arm model of e-learning, case discussion, and simulation to support all cognitive, procedural, and decision-making domains. This structure aligns with established learning theory, supports generalisability, and offers flexibility for local adaptation. Although developed in a UK context, the model is designed to be scalable and transferable, including to low-resource or multilingual settings, via low-cost simulators, offline learning, and context-specific case development.

### 4.3. Strengths and Limitations of the Review

This review has several strengths that support confidence in the findings. We followed PRISMA 2020 for study identification, screening, and selection, and a clear PICOS question guided a multi-database search across five databases [22]. Predefined inclusion and exclusion criteria kept the scope focused, omitting designs outside scope. We appraised study quality with the MERSQI tool, drawing on its published validity evidence [24,25]. As meta-analysis was not feasible due to heterogeneous educational approaches and outcomes, we used a narrative synthesis aligned with SWiM guidance to keep methods explicit and reproducible [28].

There are important limitations. Studies, interventions, outcomes, and instruments varied substantially, which prevented meta-analysis and precluded pooled effect estimates. Restricting the search to English may have introduced language bias, and focusing on 2015 onwards may have excluded earlier relevant work. We did not search the grey literature, so publication bias cannot be excluded. Taken together, these factors constrain generalisability and limit causal inference [17].

### 4.4. Implications for Practice and Future Research

Building on these findings, delivery of ENT teaching should include explicit pass standards and observed performance to enable scalability and support audit. Services should also audit adherence to local guidelines and pathways—for example, in the management of epistaxis [44] and tracheostomy care [45]—to align teaching with local needs and evaluate impact. Additionally, sites should report a small core outcome set so that results are comparable across trusts [14,15].

Future studies should use randomised or quasi-experimental designs with blinded assessors and predefined primary outcomes. Follow-up at 2–3 months should test retention and behaviour in practice, targeting Kirkpatrick levels 3–4 [16]. To address the shortfall in validity, reports should include instrument validity evidence, structured within a Kane-style validity argument [46] or a Messick framework [47].

Nationally, a standardised framework should specify minimum content, validated assessment, and a core outcome set reported by every course. Implementation should be supported by faculty development and shared resources [14,15].

## 5. Conclusions

Short, structured ENT courses improve immediate confidence and knowledge, with early but limited signals of objective performance. However, most studies are predominantly single-group and rely on self-reported outcomes, so durability and impact on professional practice remain uncertain.

Even with these limits, the consistency across designs supports wider adoption with stronger evaluation. We therefore recommend a three-arm teaching model combining e-learning (for core content), case-based discussion (for clinical reasoning), and simulation (for procedural and emergency skills), underpinned by clear pass standards, observed assessment, and a small core outcome set to enable meaningful comparison across sites. This mix supports longer-term retention and day-to-day application in on-call work.

The recommendation for the next steps is comparative trials with blinded scoring and 2–3 months’ follow-up, aligned to Kirkpatrick Levels 3–4. This will enable policy, and training leads can scale what demonstrably improves care.

## Figures and Tables

**Table 1:** Summary of included studies evaluating short, structured ENT teaching interventions (2015–2025).

Author, Year	Region	Study Design	Participants	Intervention	Comparison	Key Outcomes Measured
Jegatheeswaran et al. (2023) [29]	UK	Pre–Post	41 FY Doctors	Online videos + five-station practical session (e.g., epistaxis, PTA drainage)	Pre/Post	Confidence (seven skills), preparedness (DREEM), satisfaction
Morris et al. (2025) [30]	Wales, UK	Pre–Post	152 Junior Doctors and Allied Pros	National 1-day bootcamp (six stations, e.g., Airway, Rhinology)	Pre/Post	Confidence, knowledge (MCQ), satisfaction
Bhalla et al. (2020) [10]	UK and Australia	Two-Group Comparative	51 Junior Doctors (Sim: 38, Lect: 13)	Sim-based induction (homemade models) vs. lecture-based	Sim vs. Lecture	Confidence, anxiety, knowledge (MCQ retention)
Chin et al. (2016) [11]	Canada and USA	Pre–Post	22 ENT Residents (PGY1–2)	1-day bootcamp (cadaveric models, sim scenarios)	Pre/Post	Confidence (nine procedures), satisfaction
Swords et al. (2017) [9]	UK	Pre–Post, Single-Blinded	37 Junior Doctors	1-day bootcamp (skills + simulated scenarios)	Pre/Post, 2-week Follow-up	Confidence, blinded performance assessment, behaviour change
La Monte et al. (2023) [31]	USA	Pre–Post	47 ENT Residents (PGY1–2)	1-day workshop (three sim stations, one lecture station)	Pre/Post, 2-month Follow-up	Confidence, anxiety (by scenario), satisfaction
Dell’Era et al. (2020) [32]	Italy	Pre–Post	23 ENT Residents (PGY1–4)	2-day sim event (ten diverse stations)	Pre/Post	Confidence (six skills), satisfaction (SSES)
Giri et al. (2024) [12]	Nepal	Pre–Post	41 Medical Interns	1-day didactic workshop (No simulation)	Pre/Post	Knowledge (MCQ only)
Cervenka et al. (2020) [13]	USA	Pre–Post	45 ENT Residents (PGY1–2)	1-day bootcamp (cadaveric task trainers + scenarios)	Pre/Post	Confidence, station efficacy ratings
Alabi et al. (2022) [33]	Ireland	Pre–Post	54 Surgical Trainees	4 h bootcamp (three critical scenarios)	Pre/Post	Self-assessed competence, perceived knowledge/confidence
Smith et al. (2015) [8]	UK	RCT	38 Interns	Lecture + sim scenarios vs. Lecture-only	Between Groups	Blinded viva exam score, perception of learning

FY: Foundation Year, Pros: Professionals, Sim: Simulation, Lect: Lecture, MCQ: Multiple-Choice Question, PTA: Peritonsillar Abscess, DREEM/SSES: Validated satisfaction tool names retained for precision.

**Table 2:** Summary of methodological quality and key findings of included studies (assessed via the MERSQI tool).

Author, Year	Key Findings (Primary)	Key Findings (Secondary)	MERSQI	Strengths and Limitations
Jegatheeswaran et al. (2023) [29]	Sig. ↑ confidence (7 skills, *p* < 0.001)	DREEM median 48; 100% satisfaction and recommend	9.5	S: 100% response; validated tool (DREEM). L: Pre–post; no control; self-report.
Morris et al. (2025) [30]	Sig. ↑ confidence (*p* < 0.01) and knowledge (68.5%→96.5%, *p* < 0.01)	100% felt more confident; high satisfaction.	11	S: Large n; objective MCQ with confidence. L: Single-arm design; no follow-up.
Bhalla et al. (2020) [10]	Sim: Sig. ↑ confidence, ↓ anxiety. Lect: No Δ confidence.	Sim: Superior knowledge retention (17/20 vs. 12.3/20); positive qual themes.	12	S: Comparative design; mixed methods. L: Single institution; no instrument validity.
Chin et al. (2016) [11]	Sig. ↑ confidence for 6/9 procedures (*p* < 0.05)	93% recommend; greater gain in procedural confidence.	7.5	S: Broad trainee cohort; cadaver + scenario. L: Low response (45%); self-report only.
Swords et al. (2017) [9]	Sig. ↑ confidence (*p* < 0.0001) and blinded performance (*p* = 0.0093)	Applied skills in practice (Kirkpatrick L3); high satisfaction.	11.5	S: Blinded assessment; Kirkpatrick L3. L: No control; analysis limitations noted.
La Monte et al. (2023) [31]	Sig. ↓ anxiety, ↑ confidence for simulation stations (*p* < 0.01).	92% satisfaction; epistaxis showed ↑ anxiety/↓ confidence.	8.5	S: Internal control; longitudinal. L: Self-report; low follow-up.
Dell’Era et al. (2020) [32]	Sig. ↑ confidence all skills (*p* < 0.05)	High satisfaction (SSES: 4.5/5); cadaver station highest rated.	9.5	S: Diverse sim; validated scale (SSES). L: Pre–post; small n, self-report.
Giri et al. (2024) [12]	Sig. ↑ knowledge scores (*p* < 0.0001)	N/A	11	S: Objective knowledge focus. L: No sim; no skills/behaviour; single site.
Cervenka et al. (2020) [13]	Sig. ↑ confidence all stations (*p* < 0.05)	All stations rated highly effective; PGY-2 lacked confidence.	8.5	S: Regional cohort; multi-year bootcamp. L: Self-report only; no retention data.
Alabi et al. (2022) [33]	Sig. ↑ self-rated competence (e.g., 2/5→4/5)	92% added knowledge; 85% more confident.	7.5	S: Addresses training gap. L: Self-report only; no objective measure.
Smith et al. (2015) [8]	Sim group scored higher on blinded viva (*p* < 0.05)	Sim group: higher satisfaction (DREEM, *p* < 0.001).	13.5	S: RCT with blinded assessment (viva). L: Single centre; no retention follow-up.

Sig.: Statistically Significant, ↑/↓: Increase/Decrease, Δ: Change, Sim/Lect: Simulation/Lecture group, n: Sample size, qual: Qualitative, Kirkpatrick L3: Behavioural change in clinical practice, S/L: Strengths/Limitations.

## Data Availability

No new data were created or analysed in this study. Data sharing is not applicable to this article.

## Supplementary Materials

The following supporting information can be downloaded at https://www.mdpi.com/article/10.3390/healthcare13222886/s1. Table S1: Risk of bias assessment by study and domain. Table S2: Full MERSQI scoring by study and domain (with justifications). Supplementary File: PRISMA 2020 checklist.

## Abbreviations

The following abbreviations are used in this manuscript:

DREEM	Dundee Ready Education Environment Measure
ENT	Ear, Nose and Throat
GP/GPST	General Practitioner/General Practice Specialty Trainee
MCQ/MCQs	Multiple-Choice Question(s)
MERSQI	Medical Education Research Quality Instrument
OSF	Open Science Framework
PICOS	Population, Intervention, Comparison, Outcomes, Study Design framework
PRISMA	Preferred Reporting Items for Systematic Reviews and Meta-Analyses
RCT	Randomised Controlled Trial
SHO	Senior House Officer
SSES	Satisfaction with Simulation Experience Scale
SWiM	Synthesis Without Meta-analysis

## References

[1] Dean K.M., DeMason C.E., Choi S.S., Malloy K.M., Malekzadeh S. (2019). Otolaryngology boot camps: Current landscape and future directions. Laryngoscope.

[2] Hayois L., Dunsmore A. (2023). Common and serious ENT presentations in primary care. InnovAiT.

[3] Gundle L., Guest O., Hyland L.D., Khan A., Grimes C., Nunney I., Tailor B.V., Collaborators R.S. (2023). RecENT SHO (Rotating onto ear, nose and throat surgery): How well are new Senior House Officers prepared and supported? A UK-wide multi-centre survey. Clin. Otolaryngol..

[4] Rai A., Shukla S., Mehtani N., Acharya V., Tolley N. (2024). Does a junior doctor focused ‘Bootcamp’ improve the confidence and preparedness of newly appointed ENT registrars to perform their job roles?. BMC Med. Educ..

[5] Morris S., Owens D., Cserzo D. (2024). Learning needs of junior doctors in otolaryngology: A qualitative study. J. Laryngol. Otol..

[6] Ferguson G.R., Bacila I.A., Swamy M. (2016). Does current provision of undergraduate education prepare UK medical students in ENT? A systematic literature review. BMJ Open.

[7] Mayer A.W., Smith K.A., Carrie S. (2020). A survey of ENT undergraduate teaching in the UK. J. Laryngol. Otol..

[8] Smith M.E., Navaratnam A., Jablenska L., Dimitriadis P.A., Sharma R. (2015). A randomized controlled trial of simulation-based training for ear, nose, and throat emergencies. Laryngoscope.

[9] Swords C., Smith M.E., Wasson J.D., Qayyum A., Tysome J.R. (2017). Validation of a new ENT emergencies course for first-on-call doctors. J. Laryngol. Otol..

[10] Bhalla S., Beegun I., Awad Z., Tolley N. (2020). Simulation-based ENT induction: Validation of a novel mannequin training model. J. Laryngol. Otol..

[11] Chin C.J., Chin C.A., Roth K., Rotenberg B.W., Fung K. (2016). Simulation-based otolaryngology–head and neck surgery boot camp: ‘how I do it’. J. Laryngol. Otol..

[12] Giri S., Khan S.A., Parajuli S.B., Rauniyar Z., Rimal A. (2024). Evaluating a specialized workshop on otorhinolaryngology emergencies for junior doctors: Empowering the next generation of healers. Medicine.

[13] Cervenka B.P., Hsieh T., Lin S., Bewley A. (2020). Multi-institutional regional otolaryngology bootcamp. Ann. Otol. Rhinol. Laryngol..

[14] Association for Simulated Practice in Healthcare (ASPiH) (2023). ASPiH Standards 2023: Simulation-Based Practice in Health and Care.

[15] Health Education England (2018). A National Framework for Simulation-Based Education (SBE).

[16] Kirkpatrick D., Kirkpatrick J. (2006). Evaluating Training Programs: The Four Levels.

[17] Shadish W.R., Cook T.D., Campbell D.T. (2002). Experimental and Quasi-Experimental Designs for Generalized Causal Inference.

[18] Kruger J., Dunning D. (1999). Unskilled and unaware of it: How difficulties in recognizing one’s own incompetence lead to inflated self-assessments. J. Pers. Soc. Psychol..

[19] Miller G.E. (1990). The assessment of clinical skills/competence/performance. Acad. Med..

[20] Popay J., Roberts H., Sowden A., Petticrew M., Arai L., Rodgers M., Britten N., Roen K., Duffy S. (2006). Guidance on the Conduct of Narrative Synthesis in Systematic Reviews: A Product from the ESRC Methods Programme.

[21] Richardson W.S., Wilson M.C., Nishikawa J., Hayward R.S. (1995). The well-built clinical question: A key to evidence-based decisions. ACP J. Club.

[22] Page M.J., McKenzie J.E., Bossuyt P.M., Boutron I., Hoffmann T.C., Mulrow C.D., Shamseer L., Tetzlaff J.M., Akl E.A., Brennan S.E. (2021). The PRISMA 2020 statement: An updated guideline for reporting systematic reviews. BMJ.

[23] Ouzzani M., Hammady H., Fedorowicz Z., Elmagarmid A. (2016). Rayyan—A web and mobile app for systematic reviews. Syst. Rev..

[24] Reed D.A., Beckman T.J., Wright S.M., Levine R.B., Kern D.E., Cook D.A. (2008). Predictive validity evidence for Medical Education Research Study Quality Instrument scores: Quality of submissions to JGIM’s Medical Education Special Issue. J. Gen. Intern. Med..

[25] Reed D.A., Cook D.A., Beckman T.J., Levine R.B., Kern D.E., Wright S.M. (2007). Association between funding and quality of published medical education research. JAMA.

[26] Sterne J.A., HernÃ¡n M.A., Reeves B.C., SavoviÄ‡ J., Berkman N.D., Viswanathan M., Henry D., Altman D.G., Ansari M.T., Boutron I. (2016). ROBINS-I: A tool for assessing risk of bias in non-randomised studies of interventions. BMJ.

[27] Sterne J.A.C., Savović J., Page M.J., Elbers R.G., Blencowe N.S., Boutron I., Cates C.J., Cheng H.Y., Corbett M.S., Eldridge S.M. (2019). RoB 2: A revised tool for assessing risk of bias in randomised trials. BMJ.

[28] Campbell M., McKenzie J.E., Sowden A., Katikireddi S.V., Brennan S.E., Ellis S., Hartmann-Boyce J., Ryan R., Shepperd S., Thomas J. (2020). Synthesis without meta-analysis (SWiM) in systematic reviews: Reporting guideline. BMJ.

[29] Jegatheeswaran L., Naing T.K.P., Choi B., Collins R., Luke L., Gokani S., Kulkarni S. (2023). Simulation-based teaching: An effective modality for providing UK foundation doctors with core ENT skills training. J. Laryngol. Otol..

[30] Morris S., Burton L., Owens D. (2025). The all wales ENT SHO bootcamp: A national induction initiative. J. Laryngol. Otol..

[31] La Monte O.A., Lee J.H., Soliman S.I., Saddawi-Konefka R., Harris J.P., Coffey C.S., Orosco R.K., Watson D., Holliday M.A., Faraji F. (2023). Simulation-based workshop for emergency preparedness in otolaryngology. Laryngoscope Investig. Otolaryngol..

[32] Dell’Era V., Garzaro M., Carenzo L., Ingrassia P.L., Valletti P.A. (2020). An innovative and safe way to train novice ear, nose and throat residents through simulation: The SimORL experience. Acta Otorhinolaryngol. Ital..

[33] Alabi O., Hill R., Walsh M., Carroll C. (2022). Introduction of an ENT emergency-safe boot camp into postgraduate surgical training in the Republic of Ireland. Ir. J. Med. Sci..

[34] McGaghie W.C., Issenberg S.B., Cohen E.R., Barsuk J.H., Wayne D.B. (2011). Does simulation-based medical education with deliberate practice yield better results than traditional clinical education? A meta-analytic comparative review of the evidence. Acad. Med..

[35] Cook D.A., Hatala R., Brydges R., Zendejas B., Szostek J.H., Wang A.T., Erwin P.J., Hamstra S.J. (2011). Technology-enhanced simulation for health professions education: A systematic review and meta-analysis. JAMA.

[36] Mayer R.E. (2020). Multimedia Learning.

[37] Cook D.A., Levinson A.J., Garside S., Dupras D.M., Erwin P.J., Montori V.M. (2008). Internet-based learning in the health professions: A meta-analysis. JAMA.

[38] Liu Q., Peng W., Zhang F., Hu R., Li Y., Yan W. (2016). The effectiveness of blended learning in health professions: Systematic review and meta-analysis. J. Med. Internet Res..

[39] Ericsson K.A., Krampe R.T., Tesch-Römer C. (1993). The role of deliberate practice in the acquisition of expert performance. Psychol. Rev..

[40] Sweller J., van Merriënboer J.J.G., Paas F. (2019). Cognitive architecture and instructional design: 20 years later. Educ. Psychol. Rev..

[41] Sweller J., van Merrienboer J.J.G., Paas F.G.W.C. (1998). Cognitive architecture and instructional design. Educ. Psychol. Rev..

[42] Kolb D.A. (2014). Experiential Learning: Experience As the Source of Learning and Development.

[43] Roediger H.L., Karpicke J.D. (2006). Test-enhanced learning: Taking memory tests improves long-term retention. Psychol. Sci..

[44] Chynoweth J., Jones B.G., Stevens K. (2017). Epistaxis 2016: National audit of management. J. Laryngol. Otol..

[45] McGrath B. (2014). Comprehensive Tracheostomy Care: The National Tracheostomy Safety Project Manual.

[46] Kane M.T. (2013). Validating the interpretations and uses of test scores. J. Educ. Meas..

[47] Messick S. (1995). Validity of psychological assessment: Validation of inferences from persons’ responses and performances as scientific inquiry into score meaning. Am. Psychol..

